# Cytokine responses of immunosuppressed and immunocompetent patients with *Neoehrlichia mikurensis* infection

**DOI:** 10.1007/s00430-022-00737-6

**Published:** 2022-04-17

**Authors:** Linda Wass, Hanne Quarsten, Per-Eric Lindgren, Pia Forsberg, Elisabet Skoog, Kenneth Nilsson, Christine Lingblom, Christine Wennerås

**Affiliations:** 1 Department of Infectious Diseases, Institute of Biomedicine, Sahlgrenska Academy, University of Gothenburg, Guldhedsgatan 10A, 413 46 Gothenburg, Sweden; 2 Department of Clinical Microbiology, Sahlgrenska University Hospital, Gothenburg, Region Västra Götaland Sweden; 3 Department of Medical Microbiology, Sørlandet Hospital Health Enterprise, Kristiansand, Norway; 4 Division of Inflammation and Infection (II), Department of Biomedical and Clinical Sciences, Linköping University, Linköping, Sweden; 5 Division of Clinical Microbiology, Laboratory Medicine, County Hospital Ryhov, Jönköping, Sweden; 6 Department of Infectious Diseases, Uppsala University Hospital, Uppsala, Region Uppsala Sweden; 7 Department of Medical Sciences, Section of Clinical Microbiology and Infectious Diseases, Uppsala University, Uppsala, Sweden

**Keywords:** *Neoehrlichia mikurensis*, Neoehrlichiosis, Tick-borne disease, Immunosuppression, B cell, Cytokines

## Abstract

**Purpose:**

The tick-borne bacterium *Neoehrlichia mikurensis* causes the infectious disease neoehrlichiosis in humans. Vascular endothelium is one of the target cells of the infection. Neoehrlichiosis patients with compromised B cell immunity present with more severe inflammation than immunocompetent patients. The aim of this study was to compare the cytokine profiles of immunocompetent and immunosuppressed patients with neoehrlichiosis.

**Methods:**

Blood samples from Swedish and Norwegian immunosuppressed (*N* = 30) and immunocompetent (*N* = 16) patients with neoehrlichiosis were analyzed for the levels of 30 cytokines, using a multiplex cytokine assay and ELISA. A gender-matched healthy control group (*N* = 14) was analyzed in parallel. Data were analyzed using the multivariate method OPLS-DA.

**Results:**

The multiplex cytokine analyses generated more cytokine results than did the uniplex ELISA analyses. Multivariate analysis of the multiplex cytokine results established that increased levels of FGF2, GM-CSF, CXCL10, and IFN-γ were associated with immunosuppressed patients, whereas increased levels of IL-15 and VEGF were associated with immunocompetent neoehrlichiosis patients. When multivariate analysis findings were confirmed with uniplex ELISA, it was found that both groups of patients had similarly elevated levels of VEGF, FGF2 and IFN-γ. In contrast, the immunosuppressed patients had clearly elevated levels of CXCL10, CXCL13 and BAFF, whereas the immunocompetent patients had the same levels as healthy controls.

**Conclusion:**

Pro-angiogenic and type 1 cytokines were produced as part of the host response of neoehrlichiosis independent of immune status, whereas immunosuppressed neoehrlichiosis patients produced cytokines required for B cell-mediated defense.

## Introduction

*Neoehrlichia mikurensis* is an emerging tick-borne bacterium that can infect humans and cause neoehrlichiosis [1]. More than half of the published cases from Europe involve immunosuppressed patients who have presented with fever of uncertain cause, often in combination with thromboembolic and vascular events, such as repeated and severe thrombophlebitis, deep vein thrombosis, pulmonary embolism and transitory ischemic attacks [2]. In contrast, the clinical picture of neoehrlichiosis in immunocompetent individuals can vary from asymptomatic cases to febrile disease and even suspected fatal outcome [3–5]. Immunocompetent patients with neoehrlichiosis have presented with erythematous skin rashes in the absence of serological evidence to support a diagnosis of concomitant *Borrelia*-infection [6, 7]. We showed in a recent survey of a cohort of 40 Swedish neoehrlichiosis patients that while there was no difference in the incidence of vascular events between immunosuppressed and immunocompetent patients, the immunosuppressed ones tended to contract venous vascular events whereas the immunocompetent ones had involvement of the arterial side of the circulation [8]. *N. mikurensis* has been identified within circulating endothelial cells in the blood of patients with neoehrlichiosis, which implies that vascular endothelium is one of the targets of this infection [9].

Patients with compromised B cell immunity are susceptible to severe neoehrlichiosis. Patients at risk for grave disease are those with clonal B cell diseases, such as systemic rheumatic diseases, other autoimmune diseases and hematologic malignancies [1]. Biological agents directed against B cells, e.g., rituximab targeting CD20 on B cells, are important risk factors and are commonly used to treat multiple sclerosis patients, malignant B cell lymphomas and systemic rheumatic diseases [1, 10, 11]. Advanced age, recent chemotherapy, systemic corticosteroid treatment and splenectomy are additional risk factors for severe neoehrlichiosis [2].

*N. mikurensis* belongs to the family *Anaplasmataceae*, like the related human pathogenic bacterial species *Anaplasma phagocytophilum* and *Ehrlichia chaffeensis* [12]. However, unlike the latter two species, *N. mikurensis* has not yet been detected in North America, possibly because it has *Ixodes ricinus* as its main tick vector [2]. Due to its intracellular nature, *N. mikurensis* does not grow in blood cultures or any other cell-free media and it is consequently missed by routine microbiologic methods. At present, PCR is the sole diagnostic method available since no serological methods have been established [2].

To date, only two reports concerning cytokine responses in neoehrlichiosis patients have been published, comprising one immunosuppressed and two immunocompetent patients [7, 13]. The first case was a 77-year-old immunosuppressed individual with B cell chronic lymphocytic leukemia who exhibited increased levels of the cytokines, interleukin (IL)1β, 6, 8, 10, interferon gamma (IFN)-γ and tumor necrosis factor (TNF)-α [13]. The immunocompetent patients with neoehrlichiosis had increased levels of pro-inflammatory- and Th1 cytokines in serum, which correlated with concentrations of *N. mikurensis* DNA in serum [7]. The detected cytokines were increased levels of IFN-γ-induced protein 10 (CXCL10), IL-1β, IL-6, IL-12, IFN-γ, monocyte chemoattractant protein (MCP)-1, macrophage inflammatory protein (MIP)-1β, and TNF-α.

The objective of this study was to compare the cytokine responses in the blood of immunosuppressed and immunocompetent patients with neoehrlichiosis to increase the understanding of how immune defenses to this emerging pathogen are engaged depending on immune status.

## Materials and methods

### Study subjects

Blood samples derived from patients (*N* = 46) diagnosed by PCR with neoehrlichiosis were investigated together with samples from age- and gender-matched healthy controls (*N* = 14). Neoehrlichiosis patients were divided into two study groups, immunosuppressed (IS-Neo; *N* = 32) and immunocompetent (IC-Neo; *N* = 14). Patients were diagnosed at Sahlgrenska University Hospital, Gothenburg, Sweden (*N* = 36), Sørlandet Hospital, Kristiansand, Norway (*N* = 8) or in the Tick Borne Diseases STING study (*N* = 2) [14]. Clinical features of the study patients are listed in Table 1.

**Table 1:** Characteristics of patients with neoehrlichiosis and healthy, age- and gender- matched reference controls in the present study

Characteristic	Immunosuppressed with neoehrlichiosis (*N* = 30)	Immunocompetent with neoehrlichiosis (*N* = 16)	Healthy, uninfected (controls) (*N* = 14)	*P*-value
Mean age ± SD	63 ± 7	59 ± 10	48 ± 19	0.0011
Male *N* (%)	16/30 (53)	8/16 (50)	9/14 (64)	ns
Hematologic malignancy *N* (%)	13/30 (43)	0/16 (0)	N/A	0.0019
Systemic rheumatic disease *N* (%)	8/30 (27)	0/16 (0)	N/A	0.0230
Multiple sclerosis *N* (%)	5/30 (17)	0/16 (0)	N/A	0.0837
Hypertension *N* (%)	4/30 (13)	1/16 (6)	N/A	0.4623
Rituximab treatment *N* (%)	18/30 (67)	N/A	N/A	< 0.0001
Fever *N* (%)	29/30 (97)	3/16 (19)	N/A	< 0.0001
Vascular event *N* (%)	17/30 (57)	5/16 (31)	N/A	0.1003
Erythema migrans *N* (%)	0/30 (0)	9/16 (56)	N/A	< 0.0001

Vascular event; thrombophlebitis, deep vein thrombosis, pulmonary embolism and/or transitory ischemic attacks. Data are presented as mean ± SD for normally distributed continuous data, total number and percentage for categorical data. ANOVA was used for normally distributed continuous data and Chi-square test was used for categorical data.

*Ns* no significance, *N/A* not applicable

### Ethics statements

All participants provided written informed consent for the study. The study was approved by the local Ethical Review Boards of Gothenburg (298-05 and 2018/658) and Uppsala (2015/249), Sweden and by the Norwegian Regional Committee for Medical and Health Research Ethics, the South-Eastern region (REK ref. 204409). The STING study [14] was approved by the Regional Ethical review board at Linköping University, Sweden (M132-06)**.** All the participants provided written informed consent for the study.

### Blood samples

Plasma and serum samples isolated from venous blood collected from the patients before the administration of antibiotics (doxycycline) were analyzed, together with reference plasma and serum samples from healthy gender-matched individuals (*N* = 14). The Swedish samples were collected between the years 2009 and 2019 and were stored at − 140 °C until analysis. Samples from Norwegian patients were serum samples, stored at − 70 °C until analysis.

### Cytokine assays

The concentrations of 27 cytokines in diluted plasma and serum samples were analyzed using the fluorescence-based immunoassay Bioplex Pro™ human cytokine standard 27-plex panel (Bio-Rad Laboratory, Hercules, CA, USA) encompassing IL-1β, IL-1 receptor antagonist (IL-1RA), IL-2, IL-4, IL-5, IL-6, IL-7, IL-8, IL-9, IL-10, IL-12p70, IL-13, IL-15, IL-17, eotaxin, fibroblast growth factor basic (FGF) 2, granulocyte colony-stimulating factor (G-CSF), granulocyte macrophage colony-stimulating factor (GM-CSF), IFN-γ, CXCL10, MCP-1, MIP-1α, MIP-1β, platelet-derived growth factor-BB (PDGF-BB), RANTES, TNF-α; and vascular endothelial growth factor (VEGF). Cytokine data were analyzed using a Bio-Rad BioPlex 200 instrument equipped with BioPlex Manager software version 6.0 (Bio-Rad Laboratory). Data points that were measured as default “out of range” by the manufacturer’s software were manually determined by calculating the fluorescence intensity of each sample and comparing it with the fluorescence intensity of the standard curve, as described by Breen et al. [15]. The data sets derived from the 27-plex cytokine array are presented as the fold change of concentration for each cytokine level, relative to an average value of the healthy individuals. This was done to normalize assay-to-assay variation. Fold-change calculations were always based on data obtained in the same microtiter plate, to compensate for inter-assay variability.

Quantikine ELISA (R&D systems, Minneapolis, MN, USA) kits were used for uniplex concentration measurements of the cytokines CXCL10, chemokine (C-X-C motif) ligand 13 (CXCL13), IFN-γ, IL-15, IL-21, VEGF, GM-CSF, FGF2 and B cell-activating factor (BAFF). The serum and plasma samples were diluted 1:2 and analyzed in 96-well Half-Area Microplates (Corning, Tewksbury, MA, USA). Samples with high cytokine concentrations that were out of range were diluted and re-analyzed.

### Statistics

The multivariate method, “orthogonal projection to latent structures by means of partial least squares-discriminant analysis” (OPLS-DA), was employed, using SIMCA-P software version 15.0.2 (MKS Data Analytics Solutions, Malmö, Sweden). Generated two-component models are given a value for explanatory power or robustness of fit, R2, which estimates the amount of variance in Y that is explained by the X-variables. A high value implies that the X-variables have generated a model capable of explaining differences between study groups. Models are also given a value for stability, Q2, which is determined with cross validation, whereby one study subject is removed from the model to test the capacity of the remaining subjects to separate the study groups. This was repeated for all subjects. A high value indicates that the model is stable no matter which subject is removed.

The Mann–Whitney test was used to compare groups of two and the Kruskal–Wallis test to compare groups of three, using GraphPad Prism 8 (GraphPad Software Inc., La Jolla CA, USA). *P*-values < 0.05 were considered statistically significant.

## Results

### Defining the cytokine patterns of immunosuppressed and immunocompetent patients with neoehrlichiosis using the multiplex cytokine assay

**Fig. 1:**
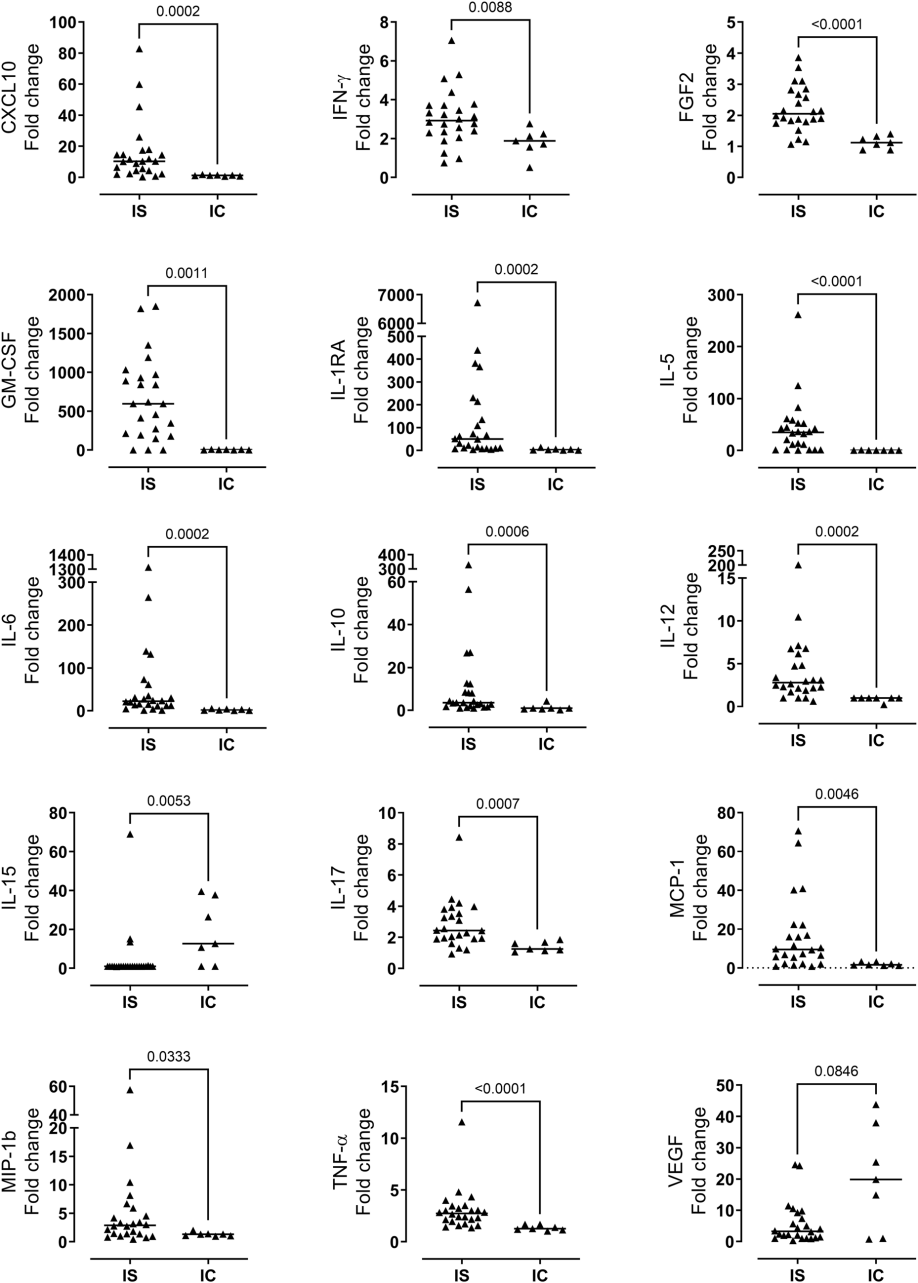
Different (*N* = 15) cytokine levels in the blood of immunosuppressed and immunocompetent patients with neoehrlichiosis determined by multiplex immunoassay. Fold changes are shown for plasma cytokine levels of immunosuppressed patients (IS, *N* = 24) and immunocompetent (IC, *N* = 7) patients in relation to the average cytokine levels in the plasma of healthy controls (HC, *N* = 10). Statistically significant differences between the groups were determined by the Mann–Whitney test. *p* < 0.05 was considered statistically significant

The levels of 27 cytokines were analyzed in the plasma of immunosuppressed patients with neoehrlichiosis (IS-Neo; *N* = 23), immunocompetent patients with neoehrlichiosis (IC-Neo; *N* = 7) and healthy controls (HC; *N* = 10). The IS-Neo group possessed higher levels of CXCL10, IFN-γ, FGF2, GM-CSF, IL-1RA, IL-5, IL-6, IL-10, IL-12, IL-17, MCP-1, MIP-1β and TNF-α, and lower levels of IL-15 compared with the IC-Neo group (Fig. 1). VEGF levels were also higher in the IS-Neo group but did not reach the statistical significance (*p* value = 0.0846).

The following cytokines were not detected or statistically significant in the fold change comparison between the samples from patients with neoehrlichiosis and the average levels of the healthy controls: Eotaxin, G-CSF, IL-1β, IL-2, IL-4, IL-7, IL-8, IL-9, IL-13 and MIP-1α. Two cytokines, PDGF-bb and RANTES, were not taken into consideration since they may leak from blood platelets if the sample is not immediately centrifuged and frozen, giving rise to potentially false-positive results [16, 17].

**Fig. 2:**
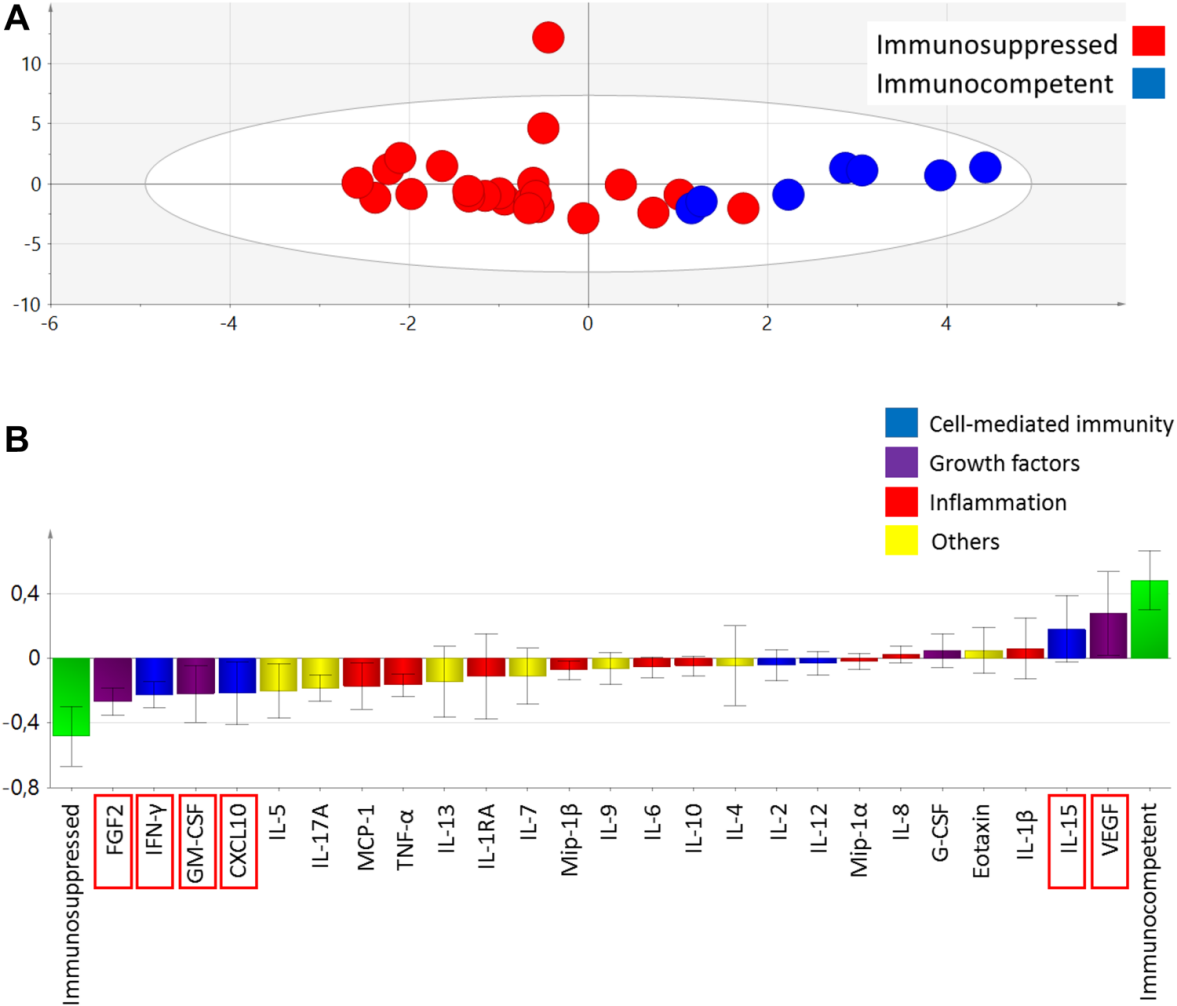
Multivariate analysis of the identification of cytokines associated with immunosuppressed- (IS-Neo, *N* = 23) or immunocompetent (IC-Neo, *N* = 7) neoehrlichiosis patients, determined in the study groups by the multiplex cytokine assay, Bioplex Pro™ human cytokine standard 27-plex panel. (**A**) “Orthogonal partial least squares-discriminant analysis” (OPLS-DA) shows segregated neoehrlichiosis study groups based on 25 cytokines. The red dots indicate IS-Neo cases and the blue dots indicate IC-Neo cases. (**B**) OPLS-DA analysis showed cytokine patterns distinguishing the IS-Neo (*N* = 23) and IC-Neo (*N* = 7) cases. Loading plots with jack knife confidence intervals for the cytokines are shown as boxes with ticks. The blue color indicates cytokines that are involved in cell-mediated immunity, purple color indicates growth factors, the red color indicates cytokines involved in inflammation, and the yellow color is for ungrouped cytokines, labeled as “Others”. The red boxes represent cytokines that were subsequently analyzed by ELISA

Using the multivariate OPLS-DA method, we constructed a model in which the study patients were set as Y-variables (Y1 for immunosuppressed and Y2 for immunocompetent) and cytokine levels (25 cytokines) were set as X-variables. The two study groups formed partly overlapping clusters and the generated two-component model (PC1 and PC2) had an explanatory power of 65% (a goodness of fit, *R*^2^*Y* = 0.65) and stability of 55% (*Q*^2^*Y* = 0.55) (Fig. 2A). Cytokines were grouped into four main categories: cell-mediated immunity, inflammation, growth factors and “other”. The cytokines that contributed to distinguishing the immunocompetent patients from the immunosuppressed patients with neoehrlichiosis are shown in a loading plot (Fig. 2B). Here, increased levels of FGF2, IFN-γ, GM-CSF and CXCL10 were associated with IS-Neo, whereas increased levels of VEGF and IL-15 were associated with IC-Neo. Therefore, these cytokines were chosen for further analyses, using the ELISA method.

### Verification of multiplex cytokine data by uniplex ELISA

**Fig. 3:**
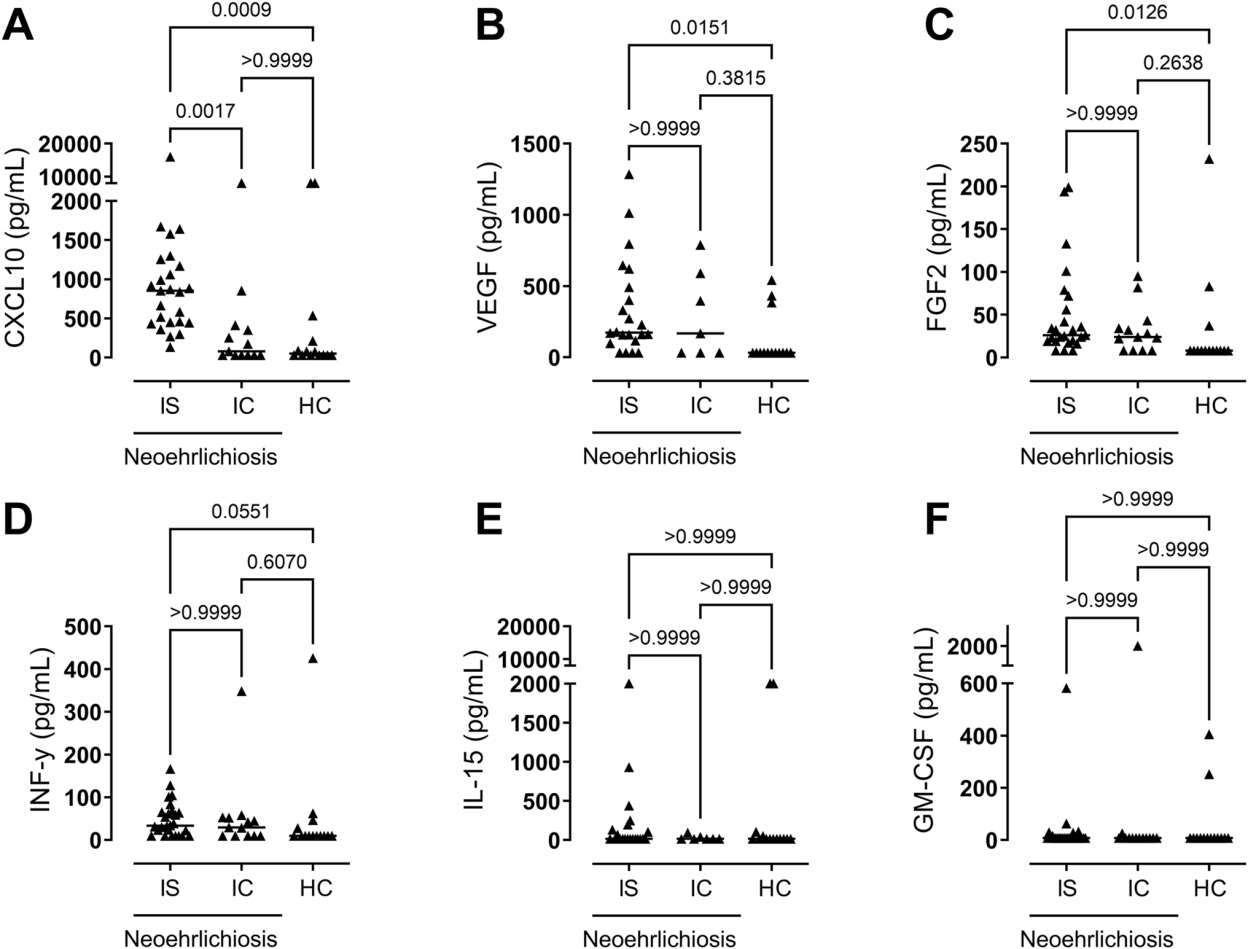
Cytokine levels in the blood of patients with neoehrlichiosis, determined by uniplex ELISA. Immunosuppressed (IS, *N* = 30) and immunocompetent (IC, *N* = 16) patients with neoehrlichiosis and healthy controls (HC, *N* = 14) were analyzed for the plasma or serum levels of (**A**) chemokine ligand 10 (CXCL10), (**B**) vascular endothelial growth factor (VEGF), (**C**) fibroblast growth factor basic (FGF2), (**D**) interferon gamma (IFN-γ) (**E**) interleukin 15 (IL-15) and (**F**) granulocyte macrophage colony-stimulating factor (GM-CSF). Statistically significant differences between the groups were determined by the Kruskal–Wallis test (**A**–**F**). *p* < 0.05 was considered statistically significant

During the course of the study, additional patients were recruited. To confirm our multiplex cytokine assay results, we chose to verify our findings by re-testing patient samples for selected cytokines using uniplex ELISA kits. The levels of FGF2, IFN-γ, GM-CSF, CXCL10, IL-15, and VEGF were measured in plasma and serum samples previously analyzed with multiplex (IS-Neo; *N* = 23, IC-Neo; *N* = 7, HC; *N* = 10) and in the samples of newly recruited patients (IS-Neo; *N* = 9, IC-Neo; *N* = 7, HC; *N* = 4). CXCL10 was clearly elevated in the blood of IS-Neo, compared with the IC-Neo group and the healthy control group (Fig. 3A). In addition, the levels of VEGF (Fig. 3B), FGF2 (Fig. 3C), and IFN-γ (Fig. 3D) were similarly raised in the blood of both the IS-Neo and IC-Neo groups compared with the healthy controls. Regarding the IL-15 and GM-CSF levels, no significant differences were observed between the two study groups or healthy controls (Fig. 3E,F).

### Additional cytokines

**Fig. 4:**
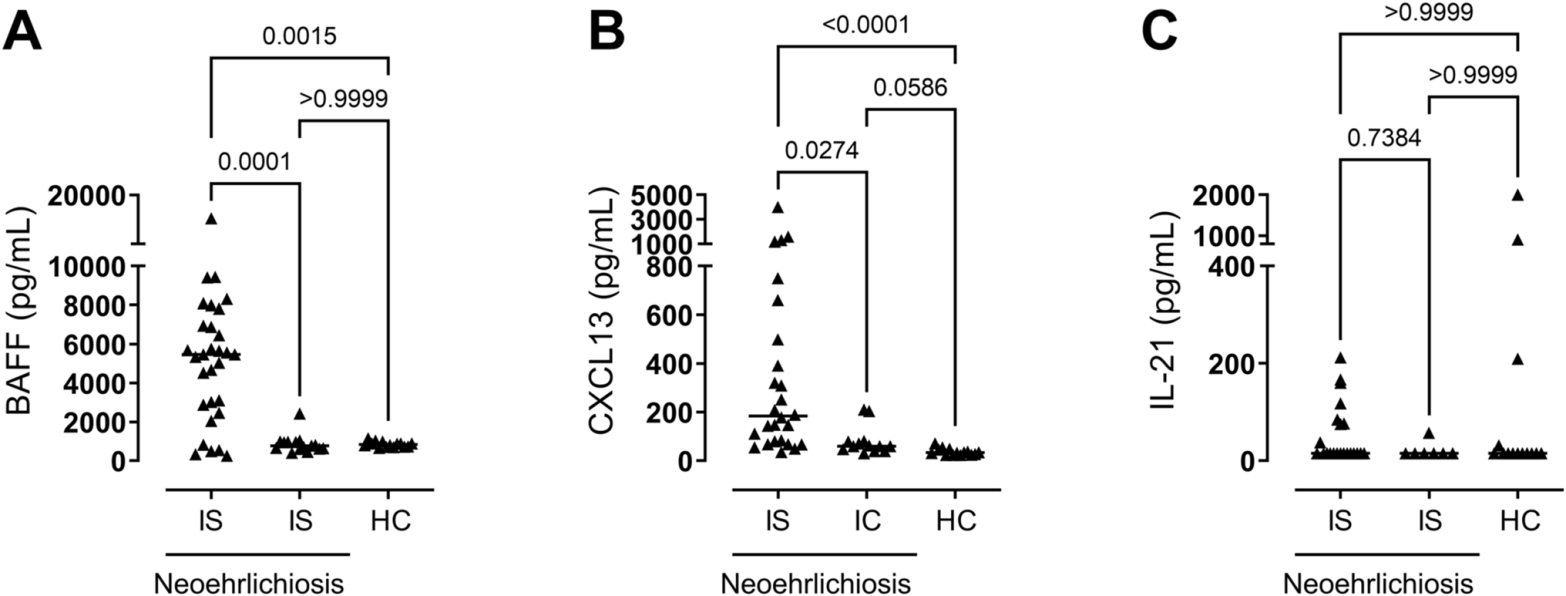
The levels of cytokines involved in B cell function in the blood serum or plasma samples of immunosuppressed (IS, *N* = 30) patients and immunocompetent (IC, *N* = 16) patients with neoehrlichiosis and healthy controls (HC, *N* = 14). (**A**) B cell-activating factor (BAFF), (**B**) chemokine ligand 13 (CXCL13) and (**C**) interleukin 21 (IL-21). Statistically significant differences between the groups were determined by the Kruskal–Wallis test (**A**–**C**). *p* < 0.05 was considered statistically significant

As most of immunosuppressed patients with neoehrlichiosis had suppressed B cell immunity (Table 1), cytokines of importance for B cell function were also analyzed, namely the levels of BAFF, CXCL13 and IL-21. A massive production of BAFF (Fig. 4A) was seen among the IS-Neo group with almost 17-fold higher levels, compared with the levels in the IC-Neo group and in healthy individuals. The IS-Neo patients also exhibited higher levels of CXCL13 than the IC-Neo patients and the healthy control group (Fig. 4B). No significant differences in IL-21 cytokine levels were observed between the study groups or healthy controls (Fig. 4C).

## Discussion

The 27-plex cytokine assay yielded more findings than the ELISA assays. Multiplex cytokine assays make it possible to screen for many cytokines using limited volumes of patient sample but have lower reliability because of the large number of capture and detection antibodies employed. Heterophilic antibodies may be present in human sera and can bind to immunoglobulins of other species, giving rise to false-positive results by bridging capture and detection antibodies, or false-negative results by sterically blocking capture antibody binding sites, or both [18]. Such antibodies may also be present in animal sera used to manufacture the immunoassays. With this in mind, we chose to first screen the patient samples for cytokine patterns using multiplex assay in combination with the multivariate method for pattern recognition, and subsequently to verify these findings by (uniplex) ELISA.

The immunosuppressed patients and the immunocompetent patients with neoehrlichiosis had similarly elevated levels of IFN-γ, VEGF and FGF2. Interferon-gamma is the prototype cytokine for cell-mediated immunity, which facilitates the inactivation of intracellular microbes by various mechanisms, one of which is to boost the microbicidal capacity of macrophages and monocytes. Raised levels of IFN-γ were also seen in the immunosuppressed and immunocompetent patient cases published by our group, albeit those results were based on 6-plex and 27-plex immunoassays, respectively [7, 13]. Since *N. mikurensis* is an intracellular pathogen, cellular immunity is likely to be necessary for host control of infection. Many closely related bacteria of *N. mikurensis*, such as *E. ruminantium* and *E. chaffeensis*, induce type 1 cell-mediated immunity and IFN-γ production in infected hosts [19–22].

The finding of increased levels of the growth factors VEGF and FGF2 is novel. We showed in 2019 that the vascular endothelium is a target of neoehrlichial infection in humans [9]. Further, more than half of patients with neoehrlichial infection have evidence of inflamed and/or damaged blood vessels as we recently reported in a cohort study [8]. It is reasonable to assume that the increased levels of VEGF and FGF2 were produced to heal injured endothelium. VEGF and FGF2 stimulate migration and proliferation of endothelial cells to generate and stabilize new blood vessels [23]. Increased serum levels of VEGF and FGF2 have earlier been reported in patients with autoimmune vascular diseases such as polyarteritis nodosa [24] and Takayasu’s arteritis [25], two conditions with clinical pictures that can be confused with neoehrlichiosis [8].

The immunosuppressed neoehrlichiosis patients had clearly elevated levels of CXCL10, CXCL13 and BAFF, whereas the immunocompetent patients had the same levels as healthy controls. CXCL10, also known as interferon-gamma-induced protein 10, is secreted by several cell types such as monocytes, endothelial cells and fibroblasts in response to IFN-γ, which itself is mediated by the IL-12 cytokine family [26, 27]. CXCL10 is also involved in promotion of T cell adhesion to endothelial cells and angiogenesis [26]. CXCL10 is also an angiostatic cytokine that can counterbalance angiogenic activities such as FGF2-induced neovascularization [28].

BAFF and CXCL13 are important factors for B cell development and chemoattraction. BAFF is mainly expressed in monocytes and stimulates proliferation and differentiation of B cells [29]. CXCL13, also known as B cell-attracting chemokine 1, is expressed by both follicular dendritic cells and germinal center T follicular helper cells in the B cell follicles [30]. The elevated levels of these two cytokines in the immunosuppressed group of patients probably depends to a large extent on the fact that the majority of the patients had compromised B cell immunity due to clonal malignant or autoimmune diseases combined with anti-B cell therapy. A study by Rosengren et al. showed that serum CXCL13 is predictive of the rate of B cell repopulation following a course of the anti-CD20 monoclonal antibody, rituximab, which 67% of the immunosuppressed patients in this study were treated with [31]. Similarly, B cell depletion brought on by rituximab infusions leads to increased serum levels of BAFF [29].

To conclude, pro-angiogenic and type 1 cytokines (INF-γ) were produced as part of the host response of neoehrlichiosis, independent of immune status, whereas neoehrlichiosis patients with compromised B cell immunity had raised levels of cytokines needed to compensate for B cell depletion.

## Data Availability

The datasets generated during and/or analyzed during the current study are available from the corresponding author on reasonable request.
